# Potential therapeutic target for polysaccharide inhibition of colon cancer progression

**DOI:** 10.3389/fmed.2023.1325491

**Published:** 2024-01-08

**Authors:** Jiawei Fan, Jianshu Zhu, He Zhu, Yinmeng Zhang, Hong Xu

**Affiliations:** ^1^Department of Gastroenterology, The First Hospital of Jilin University, Changchun, China; ^2^Department of Spine Surgery, The First Hospital of Jilin University, Changchun, China

**Keywords:** colon cancer, polysaccharide, immunity, intestinal microbiota, apoptosis

## Abstract

In recent years, colon cancer has become one of the most common malignant tumors worldwide, posing a great threat to human health. Studies have shown that natural polysaccharides have rich biological activities and medicinal value, such as anti-inflammatory, anti-cancer, anti-oxidation, and immune-enhancing effects, especially with potential anti-colon cancer mechanisms. Natural polysaccharides can not only protect and enhance the homeostasis of the intestinal environment but also exert a direct inhibition effect on cancer cells, making it a promising strategy for treating colon cancer. Preliminary clinical experiments have demonstrated that oral administration of low and high doses of citrus pectin polysaccharides can reduce tumor volume in mice by 38% (*p* < 0.02) and 70% (*p* < 0.001), respectively. These results are encouraging. However, there are relatively few clinical studies on the effectiveness of polysaccharide therapy for colon cancer, and ensuring the effective bioavailability of polysaccharides in the body remains a challenge. In this article, we elucidate the impact of the physicochemical factors of polysaccharides on their anticancer effects and then reveal the anti-tumor effects and mechanisms of natural polysaccharides on colon cancer. Finally, we emphasize the challenges of using polysaccharides in the treatment of colon cancer and discuss future applications.

## 1 Introduction

With economic development and changes in dietary habits, colorectal cancer (CRC) has become one of the malignant tumors that seriously threaten human health (1). CRC is the third most common malignant tumor and the second leading cause of cancer death (2) and there is increasing evidence that the incidence of CRC is getting younger (3). The existing treatment methods include surgery and chemotherapy (4). However, chemotherapy may lead to severe side effects including nausea, vomiting, myelosuppression, and peripheral neuropathy (5, 6), which can lead to a decrease in the quality of life for patients and unsatisfactory treatment results (7), Therefore, there is an urgent need for a new type of treatment method to overcome these existing drawbacks.

In recent years, an increasing number of research has uncovered the potential advantages of natural polysaccharides in the treatment of colorectal cancer. Natural polysaccharides are high-molecular-weight compounds formed by the linkage of multiple monosaccharide molecules through glycosidic bonds, with a diverse range of sources including plants, seaweeds, fungi, and more (8). This article specifically highlights widely studied and popular polysaccharides derived from various genera, including those from fungal species such as *Ganoderma lucidum*, *Hericium erinaceu*, *Morchella conic*, extracted from seaweeds like fucoidan, *Porphyra haitanensi*, laminarin, as well as polysaccharides extracted from plants like Yam, *Atractylodes macrocephal*, Mulberry leaf, Tea, and so on.

Polysaccharides usually have complex structures and multiple biological functions, such as antioxidant (9), antibacterial (10), anticancer (11), antidiabetic (12), and lipid-lowering properties (13). Additionally, due to their high biological compatibility and low harmful effects on the human body, natural polysaccharides have become a promising treatment option. Clinical studies have observed that *Ganoderma lucidum* polysaccharides can lead to relatively improved quality of life, and no significant toxicity was reported (14, 15). Polysaccharides play a role in multiple stages of colon cancer. Accumulative research indicates that polysaccharides can maintain the integrity of the intestinal barrier, balance the microbiota, enhance immune responses, and prevent cancer occurrence (16, 17). Moreover, they can inhibit the cell cycle, induce apoptosis in cancer cells, and regulate the expression of tumor-related proteins such as vascular endothelial growth factor (VEGF) and matrix metalloproteinases (MMPs) to counteract tumor development and spread (18, 19). Furthermore, in the advanced stages, particularly as an adjunct therapy for late-stage cancer patients, natural polysaccharides may represent a novel treatment strategy to complement surgery and chemotherapy in colorectal cancer. The combination of polysaccharides with chemotherapy drugs has the potential to enhance the anticancer effects of chemotherapy agents, reduce their toxic side effects, lower the required dosage, and improve the quality of life for patients (20, 21). These findings provide new targets for polysaccharide anti-tumor therapy and demonstrate its clinical potential.

**Figure 1:**
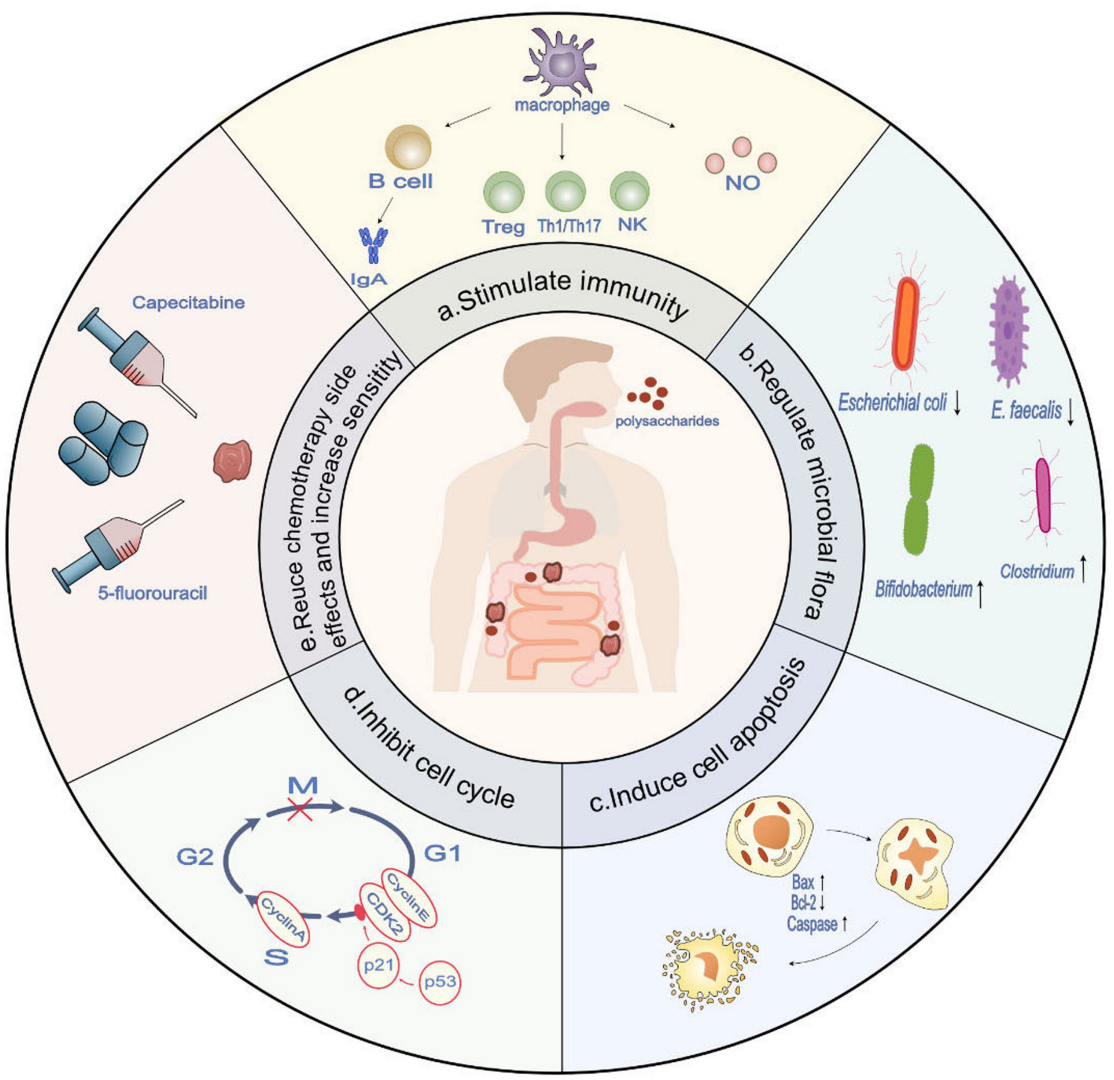
The potential role of polysaccharides in anti-colon cancer and therapeutic targets (derived from preclinical trials). (a) Polysaccharides demonstrate immune-stimulatory effects by enhancing antigen presentation in macrophages, activating T cells, B cells, and NK cells, and facilitating their differentiation towards an immune-stimulatory phenotype. This, in turn, enhances the body’s anti-tumor immune response. (b) Polysaccharides have the ability to regulate the composition of the microbiota, promoting an increase in beneficial microbiota associated with anti-colon cancer tumor immunity while reducing the presence of harmful microbiota that contribute to the incidence of colon cancer (c). Polysaccharides demonstrate the potential to induce and enhance apoptosis in colon cancer cells by modulating the Bcl-2/BAX ratio and activating the caspase family. (d) Polysaccharides have the capacity to activate p53 and upregulate p21, resulting in the decreased expression of CDK2, cyclin E, and cyclin A. This ultimately leads to cell cycle arrest specifically at the G1 and S phases. (e) Polysaccharides have been shown to reduce the side effects of chemotherapy and enhance sensitivity to treatment agents, such as 5-fluorouracil and capecitabine.

In the research involving natural polysaccharides as oral medications, we face several challenges. The large molecular weight of polysaccharides, coupled with the lack of specific enzymes in the human gastrointestinal system to break them down, limits their digestibility and therapeutic effectiveness (22). Additionally, the control of quality and consistency of polysaccharides becomes complex due to the diversity of their raw material sources (23). These factors, together with the difficulty in targeting polysaccharides to specific disease sites, may reduce their efficacy or increase the risk of side effects (24). Furthermore, interactions between polysaccharides and other food components or medications within the digestive tract can affect their release and absorption. Also, as oral medications, the palatability and patient acceptance of polysaccharides are crucial (25). In recent years, significant advancements have been made in the research of polysaccharides for the prevention and adjunctive treatment of colon cancer (26). The revelation of new mechanisms and encouraging results from clinical trials have been particularly inspiring. Despite this progress, there is a noticeable lack of comprehensive reviews synthesizing these latest findings, and assessing their effects on colon cancer treatment across various dimensions—from *in vitro* experiments and animal model studies to clinical trials. This article aims to fill this gap. We have conducted an exhaustive analysis of how different molecular structures of polysaccharides affect their functionality and delved deeply into the potential mechanisms by which these natural polysaccharides combat colorectal cancer (Figure 1). Moreover, this paper summarizes the outcomes observed in clinical trials, thereby enriching the knowledge base regarding the application of polysaccharides in colon cancer treatment. These novel discoveries not only reveal the tremendous potential of natural polysaccharides as therapeutic agents but also provide crucial insights into their prospects for practical clinical applications. Although there are several challenges in the clinical application of natural polysaccharides, we firmly believe that through in-depth research and continuous technological innovation, these limitations can be overcome, significantly enhancing the clinical application value of natural polysaccharides.

## 2 The effect of the physicochemical properties of polysaccharides on their anticancer pharmacology

The molecular size, solubility, structure, and intermolecular interactions of polysaccharides directly influence their absorption, distribution, and biological activity within the body, thereby affecting their anticancer efficacy. Variations in physicochemical factors can lead to significant changes in the biological activity and anticancer effectiveness of polysaccharides (27). Polysaccharides, large molecules formed by sugar units linked via glycosidic bonds, have structures and bond types that significantly impact their biological functions. The type (e.g., α- or β-bonds) and configuration directly influence the three-dimensional structure of polysaccharides, affecting their interactions with cancer cells and other biomolecules (28). Structures formed by α-1,4- and α-1,6-glycosidic bonds are looser, more soluble, and more easily broken down by digestive enzymes, enhancing the bioavailability of polysaccharides. Polysaccharides with β-glycosidic bonds are more stable against human enzymes and are less likely to degrade. Moreover, (1 → 3)-β-D-glucans are particularly effective in activating the immune system, especially through stimulating macrophages and natural killer cells to produce nitric oxide to combat cancer cells, thereby demonstrating antitumor activity in the treatment of colon cancer (29, 30). These glycosidic bond structures can also bind to cancer cell surface receptors, inducing cancer cell apoptosis.

Additionally, functional groups on polysaccharide molecules, such as sulfate and acetyl groups, play a crucial role in their biological activity. These groups can affect the solubility, stability, and affinity of polysaccharides for cell receptors (31). Shao et al. (32) reported the extraction of sulfated polysaccharide (SHP30) from *Sargassum horneri*, with an average molecular weight of approximately 1.58 × 10^3^ kDa, demonstrating significant antioxidant and antitumor activities in DLD cells. Selenium-conjugated modifications have shown excellent performance in promoting apoptosis, enhancing immunity, and antioxidant activities. For instance, selenium-modified soluble longan polysaccharide (SeLP) upregulates intracellular reactive oxygen species and [Ca^2+^]i levels and induces apoptosis by modulating various genes such as caspase-3/-8/-9, Bcl-2, showing stronger growth inhibition on human colon cancer HT-29 cells compared to unmodified LP (33). The antioxidant and immune activities of selenium-modified *Atractylodes macrocephala* Koidz polysaccharides are also significantly enhanced (34, 35). Furthermore, Guan et al. (36) discovered that low doses (5 μg/mL) of selenium-rich yam polysaccharides (SeYPS-1 and SeYPS-2) extracted from *Dioscorea opposita* Thunb effectively enhance the phagocytic activity of mouse spleen cells, increase the CD4^+^/CD8^+^ ratio in mouse spleen cells, and boost macrophage cytokine activity. This may be due to their stronger interactions with specific receptors on the surface of tumor cells (31). Additionally, an *in vitro* study found that the food-derived *Tremella fuciformis* polysaccharide TFP-F1 (1.87 × 10^3^ kDa) lost its ability to stimulate J774A cells to secrete TNF-α and IL-6, as well as its capacity to bind with toll-like receptor 4 (TLR4) on macrophages after removal of o-acetyl groups, confirming that functional group modifications can enhance the immunostimulatory activity of polysaccharides (37).

Molecular weight is a key factor influencing the pharmacological effects of polysaccharides, affecting their solubility, bioavailability, and interactions with cellular receptors. Polysaccharides with lower molecular weights can easily penetrate cell membranes and linger longer in the body, potentially enhancing their biological activity. Additionally, the configuration of polysaccharide chains, such as linear or branched structures, also influences their interactions with cells (38). Ma et al. (39) reported that *Tremella fuciformis* polysaccharides with low molecular weights (1,000 Da–10,000 Da) can penetrate multiple cellular membrane barriers to reach target organs and exhibit enhanced immunomodulatory activity in cyclophosphamide-treated mice. It can be inferred from previous studies that different types of natural polysaccharides may possess a variety of physicochemical properties that interact with each other in an unpredictable manner (40).

Furthermore, due to the structural diversity and variability of natural polysaccharides, obtaining their precise structures poses a significant analytical challenge (41). To enhance the anticancer potential of polysaccharides, scientists may need to optimize their bioavailability by altering their physicochemical properties, such as employing nanotechnology or chemical modifications to enhance stability, solubility, and intracellular delivery efficiency, thereby increasing the effective concentration and anticancer effects in the body. In the field of polysaccharide structural analysis, several key technologies have been widely utilized to improve analytical sensitivity and the ability to resolve complex structures. Gel permeation chromatography (GPC), particularly high-performance GPC (HPGPC) and high-performance size-exclusion chromatography (HPSEC), are commonly used methods for assessing the molecular weight and homogeneity of polysaccharides. Partial acid hydrolysis is the most frequently used method for identifying glycosidic bonds. Infrared spectroscopy (IR) identifies functional groups and glycosidic bond configurations in polysaccharides, while nuclear magnetic resonance (NMR) spectroscopy provides comprehensive structural information, including monosaccharide identification and glycosidic bond types. Liquid chromatography-mass spectrometry (LC-MS) has shown significant advances in sensitivity and specificity. X-ray diffraction (XRD) provides structural details but requires high-purity samples (38). These techniques collectively facilitate a deeper understanding of the complex structures of polysaccharides, supporting the development of polysaccharide-based drugs.

## 3 Polysaccharides strengthen the intestinal defense

### 3.1 Maintain the integrity of the intestinal epithelial barrier

The intestinal epithelial layer, serving as a crucial frontline in combating inflammation and cancer, plays a vital role. Within this layer, epithelial cells are intricately connected with factors such as Immunoglobulin A (IgA) (42), mucins (43), and short-chain fatty acids (SCFAs) (44), collectively maintaining the integrity of the gut barrier and enhancing intestinal immunity. Notably, natural polysaccharides can activate the activity of intestinal goblet cells, leading to increased mucus secretion. This mucus layer, being the first line of defense of the intestinal barrier, effectively wards off pathogens and harmful substances (45). *In vitro* experiments have shown that Cordyceps sinensis polysaccharides (CSP) and GLP promote the secretion of goblet cells and mucins, effectively reversing the downregulation of ZO-1 protein expression and upregulating the levels of tight junction proteins (TJ proteins) in colon tissue (16, 46). Moreover, research has revealed the significant role of Rhizoma *Atractylodis macrocephalae* polysaccharides (RAMPtp) in combating DSS-induced challenges. RAMPtp induces the production of a novel long non-coding RNA (lncRNA) ITSN1OT1, which prevents the phosphorylation of STAT2 nuclear input, thereby avoiding the downregulation and structural damage of TJ proteins caused by DSS, effectively maintaining intestinal barrier function (47). The preservation of these protein structures is crucial for preventing harmful substances and pathogens from permeating through intercellular gaps. *In vivo* studies by Yang et al. (48) revealed that polysaccharides extracted from *Ganoderma lucidum* significantly raised the levels of mucin, cecal propionate, and IgA, while markedly reducing the concentration of lithocholic acid in mouse feces. This finding indicates that *Ganoderma lucidum* polysaccharides (GLP) can effectively regulate the concentrations of these key substances, thereby positively impacting the intestine.

Conversely, factors such as endotoxins (49), lipopolysaccharides (LPS) (50), and secondary bile acids (BAs) (51) are considered potential risks in disrupting the integrity of the intestinal barrier and exerting cytotoxic effects on cells, thereby becoming potential risk factors for cancer. Studies have shown that administering curcumin, barley β-glucan, and *Ganoderma lucidum* polysaccharides effectively reduces the levels of secondary bile acids in the intestine (48, 52). This observation is particularly significant because secondary bile acids, such as lithocholic acid and deoxycholic acid, are cytotoxic substances in the by-products of intestinal microbiota. Deoxycholic acid can induce DNA damage through the activation of nuclear factor-kb, thereby increasing the risk of cancer (51).

Ulcerative colitis (UC) is a disease associated with weakened intestinal epithelial barrier function and increased intestinal permeability. In a dextran sulfate sodium (DSS)-induced acute colitis mouse model, ginger polysaccharide (GP) was found to regulate intestinal inflammation by inhibiting pro-inflammatory cytokine levels, modulating occludin-1 and ZO-1 protein expression, repairing the intestinal barrier, and alleviating UC symptoms (53). Additionally, *Nostoc commune* Vaucher polysaccharide was observed to enhance the gut microbiota-mediated intestinal mucosal barrier, thereby providing protective effects against UC (54). A clinical study has demonstrated that the addition of yeast polysaccharides significantly increased villus height (0.1%, 0.2%, and 0.3%) and muscle layer thickness (0.2%) (*p* < 0.05). Furthermore, the addition of 0.1% and 0.3% yeast polysaccharides significantly increased the number of goblet cells (*p* < 0.05). However, it should be noted that this experiment was conducted on channel catfish, and further human trials are needed to substantiate the effects of polysaccharides (55).

### 3.2 Strengthen the immunity of intestinal epithelium

First and foremost, our focus lies on the immune activity of polysaccharides, including *Ganoderma lucidum* polysaccharide (18), Mulberry leaf polysaccharide (56), Yam polysaccharide (57), Fucoidan (11), *Hericium erinaceus* polysaccharide (58), Ginkgo polysaccharide (59), and Tremella polysaccharide (39) have been reported *in vitro*. Non-starch polysaccharides can be consumed orally and subsequently undergo breakdown by the acidic environment and enzymes present in the digestive tract, enabling them to penetrate the epithelial cells of the small intestine. From there, they are transported via the bloodstream to the target organ (60). Following processing by the mononuclear phagocyte system, the active components of these polysaccharides are exposed, leading to the activation of Toll-like receptors and other immune receptors that interact with intestinal immune cells, such as intestinal epithelial cells and intestinal innate immune cells (61, 62). This stimulation triggers both humoral and cellular immunity in the host. Although the absorption rate of this process is low, this process holds great significance as it elucidates how the modulation of the ecological balance in the digestive system can impact the human immune system.

*In vitro* experiments have demonstrated that GLP exhibit significant immunomodulatory effects. GLP can activate T lymphocytes, B lymphocytes, and natural killer cells (NK), stimulating the production of cytokines such as TNF-α, IFN-γ, and IL-1β, while concurrently reducing the levels of IL-8 and IL-6 (63). Moreover, GLP activates B cells via the TLR2/4 receptors and initiates immune responses through receptors on antigen-presenting cells, including NKs, CR3, monocytes, macrophages, and dectin-1 (64). Additionally, the immune modulation of HEP in the fermentation broth of *Hericium erinaceus* polysaccharides has been found to play a significant role in regulating colonic immunity (65).

At a concentration of 20.00 μg/mL, GLP exhibited maximal stimulation of macrophage proliferation. Furthermore, a GLP concentration of 40.00 μg/mL significantly enhanced the production of nitric oxide (NO) (63), potentially via signaling pathways such as PI3K, p38, and MAPK (66). This is crucial for eradicating pathogens and tumor cells. Additionally, high-molecular-weight galactomannan polysaccharides, isolated from the edible mushroom *Morchella esculenta*, with a molecular weight of approximately 1 million Da, showed notable biological activity. At a concentration of 3.0 μg/mL, this galactomannan maximally activated NF-kappa B and enhanced macrophage expression, thereby stimulating NO production (67).

Similarly, sulfated polysaccharides derived from *Ceryx fulvescens* (68) and the *Rhizopus nigricans* polysaccharide (RPS-1) from the Zygomycete filamentous fungus have been effective in activating macrophages (69). RPS-1, in particular, induced NO (at 200 and 400 μg/mL) and TNF-α (50–400 μg/mL) production in RAW 264.7 macrophage cells in a concentration-dependent manner and could stimulate via MAPKs and NF-κB signaling pathways. Furthermore, oral administration of RPS-1 significantly slowed tumor growth in CT26 tumor-bearing mice (69), indicating the potential of polysaccharides in immune regulation and anti-tumor therapy.

In ongoing *in vivo* studies, we observed that 400 μg/mL of water-soluble polysaccharides from wild *Morchella conica* (MCP) significantly restored the spleen weight in mice and increased the number of white blood cells and lymphocytes in the spleen. Compared to the control group, the MCP-treated mice showed elevated levels of bacteria producing short-chain fatty acids (SCFAs) in their intestines, particularly the growth of *Ruminococcaceae* family (70). Additionally, MCP treatment led to increased production of butyrate and decreased production of acetate in the mouse intestines. Butyrate, apart from being an energy source for colon cells, enhances intestinal barrier function, reduces inflammation levels, and may consequently affect the composition of the gut microbiome (71). This helps in understanding how polysaccharides modulate immune functions.

Further experimental results demonstrate that the mulberry leaf polysaccharide MLP-2, at a concentration of 250 μg/mL, significantly promotes the proliferation of splenic lymphocytes in mice. Notably, acute toxicity tests reveal that MLP-2 poses no acute toxicity to mice, underscoring the safety of polysaccharides in immunomodulation (56). *In vitro* experiments further reveal that MLP-2 not only significantly enhances the proliferation of cecal B lymphocytes and elevates the levels of interleukin-2 (IL-2) and interferon-γ (IFN-γ), thereby mediating cellular immunity, but also promotes the production of cecal IgA cells. These alterations further augment the secretion of secretory IgA (sIgA) (56), thereby enhancing the protective function of the intestinal mucosa. IgA, a predominant antibody in the intestinal mucosa, specifically recognizes and binds to microbes in the gut, aiding in controlling their growth and activity and thus maintaining the balance of the microbiota. Significantly, IgA antibodies play a crucial role in maintaining the balance between the human body and gut flora, thereby being pivotal in intestinal immunity (72). Rolenske et al. (73) found in a mouse model that IgA antibodies can specifically limit the fitness of commensal bacteria at multiple levels, enabling the immune system to recognize and restrict the growth of these bacteria, effectively preventing their harmful effects on the intestines and the body. Intestinal immune cells and epithelial cells, such as Paneth cells, actively maintain a delicate balance of the gut microbiota by releasing various antimicrobial peptides, such as α-defensins, RegIIIγ, etc. This regulatory mechanism plays a vital role in preventing diseases associated with dysbiosis of the intestinal microbial composition (74–76). These findings provide a deeper understanding of the mechanisms by which polysaccharides modulate immune responses and promote intestinal health.

The research findings demonstrate that polysaccharides exert a notable impact on cancer cells, with their anticancer effects varying according to dosage. Additionally, they recognize that individual biological variability and specific health conditions significantly influence the efficacy of polysaccharides. These insights emphasize the necessity of adopting personalized medical strategies in cancer treatment, highlighting the importance of tailoring individual dosages, biological characteristics, and health conditions to provide more effective and safer therapeutic options. These research not only offers a novel perspective in cancer treatment but also lays the foundation for the implementation of more precise and personalized treatment strategies.

### 3.3 Regulate the intestinal microbiota

Abundant clinical research data strongly indicates a close correlation between the gut microbiota and the overall health and prognosis of patients, especially regarding a range of chronic diseases including obesity (77), diabetes (78), inflammatory bowel disease (79), and colorectal cancer (80). The gut microbiota is a highly complex system comprising over a thousand microorganisms, with *Bacteroidetes* and *Firmicutes* accounting for 90% of its composition. In patients with colorectal cancer (CRC), pathogenic bacteria such as *Escherichia coli*, *Enterococcus faecalis*, *Fusobacterium nucleatum*, and *Streptococcus gallolyticus* are more abundant, while probiotic bacteria such as *Bifidobacterium*, *Clostridium*, *Faecalibacterium*, and *Roseburia* are less abundant (81). This highlights the potential of maintaining a healthy gut microbiota for preventing and treating colon cancer.

Following that, we delved into the findings of our research regarding the impact of *Ganoderma lucidum* polysaccharides and *Flammulina velutipes* polysaccharides on the microbiota. Recent studies have indicated that these polysaccharides have the potential to enhance the diversity of the microbiota, notably elevate the proportion of *Firmicutes*, reduce the prevalence of *Bacteroides*, and foster an increased abundance of beneficial probiotics like *Lactobacillus*, *Trichomyceae*, and *Roseburia* (17, 82). Then, we performed a comprehensive review of *in vitro* experimental data. These findings showcased the consumption and probiotic activity of agarotriose AgaDP3, a jelly-derived sugar from red algae, by the HMO probiotic *Bifidobacterium*. Additionally, another hydrolysis product of agarohexose, D-galactose, and 3,6-anhydro-L-galactose (AHG), demonstrated significant inhibition of proliferation and activity in HCT-116 human colon cancer cells, leading to apoptosis in this cell (83). GLP can also alleviate microbiota dysbiosis induced by AOM/DSS by inhibiting TLR4/MyD88/NF-κB signaling (84).

Short-chain fatty acids (SCFAs), crucial metabolites of the gut microbiota, play a pivotal role in protecting the intestinal barrier. They achieve this by reducing gut pH, inhibiting pathogenic microbes, enhancing the production of mucins and mucus by epithelial cells, and upregulating tight junction (TJ) proteins (85–87). SCFAs regulate the expression of antimicrobial peptides in intestinal epithelial cells through the mTOR and STAT3 pathways activated by GPR43. They also interact with receptors such as FFA2, FFA3, and GPR109A, playing a significant role in modulating the inflammatory responses of various immune cells, including neutrophils, macrophages, and T cells, in the homeostasis of the gut microbiome (88). Observations in water-soluble polysaccharides extracted from wild morels (70), polysaccharides from *Ganoderma lucidum* (84), *Atractylodes macrocephala* polysaccharides (89) show changes in gut microbiota composition, increased SCFA production, thereby impacting gut immunity.

On the other hand, the gut microbiota can induce intestinal epithelial cells to secrete chemokines, attracting immune cells to the intestinal mucosa (90). This, in turn, promotes the quantity and distribution of intestinal immune cells (91). Numerous *in vivo* studies have shown that pathogenic bacteria can directly or indirectly induce intestinal dendritic cells to release pro-inflammatory cytokines like IL-6 and TNF (92), promoting the differentiation of intestinal CD4^+^ T cells into Th1, Th17 cells, and Tregs (93). This influences the homeostasis and sensitivity of the intestinal immune system. Therefore, significant differences in colonic microbiota composition between colorectal cancer patients and healthy individuals can reflect the state of the immune response in these patients.

Clinical data studies have indicated a close relationship between intestinal immune responses and the microbiota, with their interaction playing a crucial role in maintaining intestinal immune homeostasis and combating the onset and progression of colorectal cancer (94). Numerous clinical trials are ongoing to understand the impact of the microbiota on the development of CRC and to identify more effective treatment strategies (84). In CRC treatment, prebiotics and probiotics, as microbiota modulators, play a significant role. They are capable of inhibiting epithelial cell proliferation, reversing DNA damage, and reducing complications from surgery or chemotherapy (95). A systematic review involving 23 randomized controlled trials (RCTs) showed that supplementing CRC patients with probiotics, including Bifidobacteria and Lactobacilli, improves quality of life, reduces postoperative infections, inhibits the production of pro-inflammatory cytokines, and alleviates chemotherapy side effects, thereby improving surgical outcomes and reducing mortality risk (96). Another randomized controlled trial assessed the long-term efficacy of polysaccharide K (PSK) combined with 5-fluorouracil therapy after radical resection of CRC. The study found that polysaccharide K significantly improved patient survival rates (97). These findings offer hope for polysaccharide treatment in CRC; however, more clinical trials are needed to confirm the effects of various polysaccharides in humans. The broad regulatory roles of these polysaccharides suggest their potential as novel therapeutic agents. Further research into the specific effects of these polysaccharides on the gut microbiota and their interactions with the host immune system is essential.

## 4 Effect of polysaccharides on colon cancer progression

### 4.1 Cell cycle inhibition induced by polysaccharides

The cell cycle encompasses four distinct phases: the mitotic phase (M phase), the G1 phase preceding DNA synthesis, the S phase involving DNA synthesis, and the G2 phase preceding mitosis (98). This cycle serves as the foundation for cell growth, division, and programmed cell death, playing a pivotal role in the effectiveness of various drug therapies. Researchers have employed diverse experimental approaches to unravel the intricate regulatory mechanisms governing the cell cycle.

*In vivo*, studies have revealed the critical involvement of polysaccharides in cell cycle regulation through the Ras/ERK and p53 pathways (99). Notably, the Ras/ERK signaling pathway actively participates in cell cycle regulation and is directly linked to the transition from G1 to S phase (100). In the context of the intestine, polysaccharides are metabolized into short-chain fatty acids and other metabolites. These metabolites can bind to cell surface receptors, initiating signaling cascades in apoptotic pathways, thereby finely regulating the progression of the cell cycle and promoting cell differentiation. Gaining a comprehensive understanding of these intricate internal mechanisms not only contributes to our knowledge of cell cycle regulation but also unveils potential targets for drug development. Through *in vitro* studies, we can further explore and validate the aforementioned findings. A study conducted by Lu et al. (101) revealed that GLPs have the ability to induce cell cycle arrest in the G1 phase by blocking the ERK1/2 or ERK5 pathways, as well as inhibit cells in the G2/M phase. Moreover, GLPs can induce cell cycle arrest by inactivating cyclin-dependent kinase 1 (CDK1) and increasing the phosphorylation of tyrosine 15 (Y15). This *in vitro* research sheds light on the impact of GLPs on cell cycle regulation and provides additional support to the results obtained from *in vivo* studies (101).

Overall, the combination of *in vivo* investigations, and *in vitro* studies has unveiled the intricate mechanisms involved in cell cycle regulation. Polysaccharides and other molecules exert their influence on the cell cycle by affecting crucial signaling pathways such as Ras/ERK and p53, thus providing valuable insights and new avenues for drug development targeting the cell cycle. Future research should delve deeper into these regulatory mechanisms in order to enhance our understanding and advance disease treatments for the betterment of human health. Specifically, p53, a central tumor suppressor, plays a pivotal role in governing the cell division cycle. Upon activation, p53 strongly stimulates the transcription of p21/CDKN1A as a direct target (102). Subsequently, the kinase inhibitor p21 impedes the activity of various cyclin-CDK complexes, leading to the downregulation of multiple target gene expressions. This cascade results in the hypophosphorylation of RB, facilitating the formation of the RB-E2F complex and its binding to E2F sites in target promoters, ultimately culminating in cell cycle arrest (103). On the other hand, polysaccharides such as *Cordyceps cicadae* polysaccharide (CCP) have demonstrated their capacity to regulate the cell cycle. CCP is derived from a complex formed by fungi that parasitize cicada larvae. Treatment with CCP polysaccharide has been shown to downregulate the expression of cyclin E, cyclin A, and CDK2 while upregulating the expression of p53 (104). In another study, it was discovered that GLP has the ability to reactivate mutant p53 in colorectal cancer cells and induce cell cycle arrest (82). These findings hold promise for the development of novel therapeutic approaches targeting tumor cells and underscore the significant value of *in vitro* experiments in investigating cell cycle regulation.

Although the precise mechanisms through which certain polysaccharides inhibit the cell cycle have not been fully elucidated, clinical studies have revealed their significant role in cancer treatment. Polysaccharides not only impede the progression of the cancer cell cycle but also offer the potential for expanding the range of polysaccharide targets, thereby providing more opportunities for clinical therapy. *In vivo*, research data enables us to delve deeper into this phenomenon. Liu et al. (105) successfully isolated and purified a novel polysaccharide, HEFP-2b, with a molecular weight of 3.252 × 10^4^ Da from the fruiting body of *Hericium erinaceus*. They discovered that HEFP-2b can induce cell cycle arrest in the S phase of colon cancer cells (HCT-116). This finding further reinforces our belief in the involvement of polysaccharides in cancer treatment, particularly colorectal cancer, which is a prevalent form of cancer. Additionally, ginger polysaccharide (GP) has exhibited similar anticancer effects. GP, enzymatically extracted from ginger, contains L-rhamnose, D-arabinose, D-mannose, D-glucose, and D-galactose. Researchers have found that GP induces apoptosis by blocking the G0-G1 phase of cancer cells (99). Another *in vitro* study demonstrated that treatment with fucoidan can cause cell cycle arrest in the sub-G1 phase, affecting the cell cycle and displaying anticancer activity against densely packed HT-29 cells (106).

### 4.2 Apoptotic signaling pathway activation induced by polysaccharides

The apoptotic signaling pathway stimulated by polysaccharides has been recognized as a crucial target for the development of anticancer drugs. Although the clinical research on these mechanisms remains incomplete, we have gained insight into the notable role of polysaccharides in inducing apoptosis in cancer cells and inhibiting tumor growth and metastasis. Although this field of study is still in its early stages, several studies have indicated that polysaccharides may exert inhibitory effects on cancer cells by modulating apoptotic and autophagic pathways, as well as promoting the generation of reactive oxygen species (ROS). Mitochondria play a significant role in regulating various cell death pathways, including apoptosis, autophagy, and ROS production (107). Eukaryotic cells primarily rely on the intrinsic apoptotic pathway, which is regulated by pro- and anti-apoptotic genes, and the extrinsic apoptotic pathway, which is regulated by death receptors. The intrinsic pathway is triggered by the reduction of the Bcl-2/BAX ratio, leading to the formation of pores on the outer membrane of mitochondria through homologous oligomerization. This process releases cytochrome C (CytC) into the cytoplasm, initiating the cascade reaction of the Caspase family and ultimately inducing cell apoptosis (108). The extrinsic pathway, also known as the death receptor pathway, is activated when death receptors, such as DR3, DR5, Fas, and TNF-R1, bind to their ligands, such as FasL, through the death receptor domain (DD) and adapter proteins (FADD and TRADD) to form a signal complex (DISC). This complex further activates the Caspase family, leading to apoptosis (109).

*In vitro*, research data further elucidates the influence of polysaccharides on apoptotic signaling pathways. For example, *Ganoderma lucidum* (LingZhi) GLPs enriched with selenium have been shown to increase the activity of caspase-3/-8/-9 and PARP in a time-dependent manner, indicating their potential to trigger the death receptor signaling pathway in LoVo cells (110). *In vivo*, research results have demonstrated that polysaccharides have regulatory effects on various apoptotic signaling pathways. For instance, *Hericium erinaceus* polysaccharides (HEFPs) can regulate the expression of Bcl-2 family members (Bax and Bcl-2) and activate the caspase-9-dependent intrinsic mitochondrial pathway through MMP collapse, inducing apoptosis in HCT-116 and DLD1 cells (111). Recent studies have also highlighted HEFPs can also activate the extrinsic apoptotic pathway (TRAIL, Fas-L) and downregulate the expression of Bcl-2 and Bcl-XL in AGS cells (human gastric adenocarcinoma cells) while stimulating the histone H3K4me3 binding of FasL and TRAIL. Additionally, HEFPs can participate in AGS cell apoptosis through the ROS-derived promoter and AKT/FAK/PAK1 pathway, which broadens the understanding of tumor cell apoptosis and highlights the importance of ROS in the process (112). These findings enhance our confidence in polysaccharides as a potential strategy for cancer treatment, particularly for prevalent cancer types such as gastric and colorectal adenocarcinomas. For instance, it is currently understood that polysaccharides have the ability to induce cancer cell apoptosis by modulating both the intrinsic and extrinsic apoptotic pathways. However, the precise mechanisms through which polysaccharides exert their effects on these pathways remain unclear. This likely involves the regulation of cellular signaling transduction pathways, gene expression, protein interactions, and other levels of cellular regulation. As a result, further in-depth exploration is necessary for future research to elucidate the intricate mechanisms by which polysaccharides operate.

ROS, an important signaling molecule, is often increased in cancer cells due to hypoxic microenvironments. It can activate oncogenes, inactivate tumor suppressors, and prevent DNA repair (113). However, the role of ROS in cancer is dual, as excessive ROS can promote cell death, while low levels of ROS play an important role in promoting tumor growth (114). Hence, achieving a delicate balance in the dual role of reactive oxygen species (ROS) presents a major challenge in clinical treatment. Given that oxidative damage has been proven to trigger cell apoptosis, targeting ROS holds promise as an effective therapeutic strategy.

Mitochondria are the primary source of ROS within cells, and play a crucial role in the cellular processes, particularly in inducing cell apoptosis (115). In a previous study, HEEPs were found to increase ROS levels and induce apoptosis in HCT-116 and DLD1 cells, in a dose-dependent manner, upregulating cleaved-caspase-9 and cleaved-caspase-3. The ability of HEFPs to trigger the mitochondrial apoptotic pathway was reduced when antioxidant was added, indicating the role of ROS in the upstream pathway of HEFPs-induced apoptosis through oxidative stress (111). *In vitro*, studies also support the significance of reactive oxygen species (ROS) in anticancer mechanisms. *Porphyra haitanensis*, a widely distributed red alga, was found to increase ROS levels in human colon cancer HT-29 cells treated with *Porphyra haitanensis* polysaccharides (PHPs), leading to oxidative stress that caused damage to nucleic acids, proteins, and lipids, ultimately resulting in cell death (116). In addition, fucoidan from *Sargassum fusiforme* was shown to effectively induce apoptosis and inhibit cancer in the colon cancer cell line Caco-2 by enhancing ROS production and altering mitochondrial membrane integrity (117). A molecular study of the polysaccharide RTFP-1, derived from *Rosa roxburghii* Tratt fruit, has revealed that RTFP-1 activates apoptosis in HepG2 cells through ROS-mediated MAPK, STAT, and p53 apoptotic pathways (118). This finding potentially elucidates the underlying mechanism by which polysaccharides induce ROS.

Lysosome-induced apoptosis is a significant form of cell death, playing a critical role in maintaining intracellular homeostasis and controlling pathological states. This apoptosis mechanism primarily involves lysosomal dysfunction, leading to the release of cellular digestive enzymes such as cathepsin D, which trigger the apoptotic program in cells. This process is particularly important in preventing cancer development, regulating immune responses, and eliminating damaged or aged cells (119). Recent studies have demonstrated the importance of lysosome-induced apoptosis in cancer therapy. Zhou et al. (120), through clinical research, explored the effects of tea polysaccharides (TPs) on CT26 colon cancer cells. The results indicated that 800 μg/mL of TPs significantly increased the rate of cell apoptosis. *In vitro* studies found that TPs activated transcription factor EB (TFEB), reduced the activity of cathepsin B, leading to lysosomal dysfunction, thereby releasing lysosomal proteases, and induced apoptosis by regulating the expression of Bax1 and Bcl-2 and reducing mitochondrial membrane potential through the activation of the caspase cascade. Furthermore, in recent research, *Agrocybe cylindracea* polysaccharide (ACP) significantly inhibited the proliferation of colorectal cancer cells, with the half-maximal inhibitory concentration (IC50) against HCT-116 (and HT29) cells being 490 μg/mL (786.4 μg/mL) within 24 h. ChIP-sequencing revealed that ACP increased histone H3 lysine 27 acetylation (H3K27ac) at the cathepsin D (CTSD) promoter in HCT-116 cells, promoting the binding of transcription factor EB (TFEB), thereby triggering mitochondrial-mediated apoptosis. These findings reveal the potential of polysaccharides to combat colorectal cancer through the lysosome-mitochondria pathway, offering new mechanistic insights for future cancer treatments (121).

Autophagy is a highly conserved cellular process that plays a crucial role in maintaining homeostasis in the body. Its role in the onset and progression of cancer is complex and controversial (122). While autophagy plays a key role in protecting cancer cells from genotoxicity and metabolic stress, leading to tumor development, it is often found to be restricted in many cancers (123). Lentinan (SLNT), a low-toxicity polysaccharide extracted from Shiitake mushrooms and composed of D-glucose units, inhibits tumor growth directly by inducing autophagy and apoptosis in HT-29 tumor-bearing NOD/SCID mice. *In vivo* clinical studies have confirmed that tumor sections treated with Shiitake polysaccharide exhibit stronger BIP and CHOP fluorescence intensities (124), indicating that upstream endoplasmic reticulum stress (ERS) in autophagy and apoptosis is significant. Prolonged ERS induces cell death by activating cytotoxic autophagy and disrupting Ca^2+^ homeostasis through specific signaling pathways like PERK/ATF4/CHOP, IRE1α, etc. Additionally, another study found that *Rosa rugosa* polysaccharide RRP promotes the accumulation of autophagosomes by inducing autophagy-mediated apoptosis in human cervical cancer cells (HCCCs) through inhibition of the PI3K/AKT/mTOR signaling pathway (125). Thus, autophagy is a complex biological process whose role in cancer cells may depend on specific biological environments and signaling pathways.

ErbB2, part of the epidermal growth factor receptor (EGFR) family, has downstream signaling pathways like PI3K/Akt, MAPK (ERK1/2) closely associated with cell growth, division, and survival. Inhibition of ErbB2 signaling not only negatively impacts cell proliferation but also regulates autophagy and apoptosis proteins, promoting cell death (126). Laminarin, a polysaccharide from brown algae, was found in an *in vitro* study to effectively inhibit the phosphorylation and expression of ErbB2 in HT-29 cells (127), thus promoting cancer cell apoptosis and enriching the potential mechanisms of polysaccharides. Furthermore, *in vitro* research has revealed that brown algae polysaccharides combined with vitamin C can induce nuclear degeneration, enhancing the inhibitory effect on the viability of HCT-116 colon cancer cells (128). This discovery provides valuable new insights into how fucoidan polysaccharides affect cancer cells in an *in vitro* environment.

**Figure 2:**
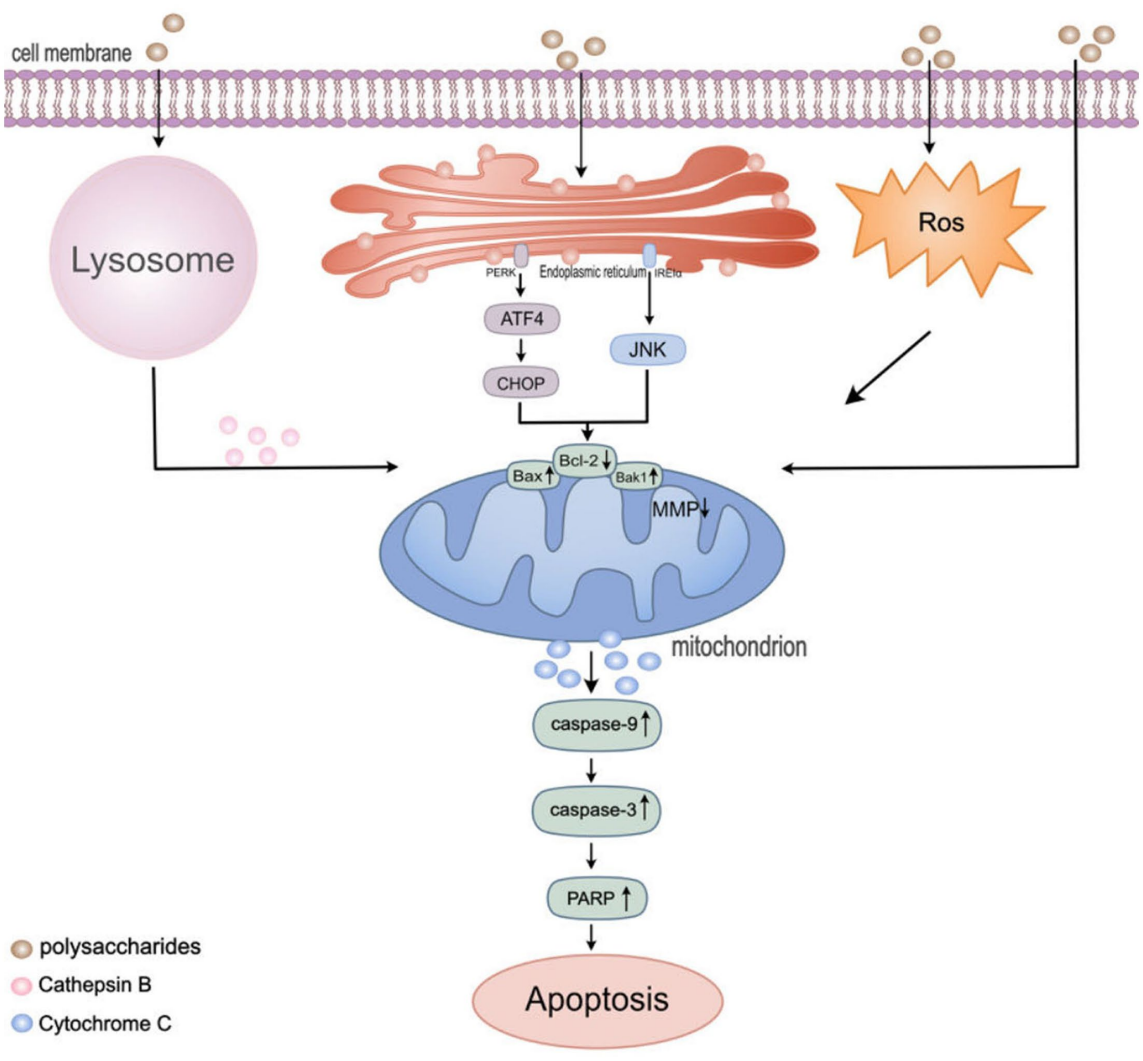
Regulation of apoptosis pathway in colon cancer cells by polysaccharides. Mitochondria play a significant role in regulating various cell death pathways, including apoptosis, autophagy, and ROS production. HEEPs were found to increase ROS levels. TPs were found to induce the release of tissue proteases from lysosomes. Lentinan, inducing autophagy and endoplasmic reticulum stress (ERS), following ERS stimulation, the PERK and IRE1α sensors located in the endoplasmic reticulum membrane dissociate from BIP and activate downstream signals such as ATF4, CHOP and JNK. Then, the level of pro-apoptotic protein Bak1 and Bax1was up-regulated and the level of anti-apoptotic protein Bcl-2 was down-regulated and caused a decrease in the mitochondrial membrane potential. Subsequently activate the caspase cascade reaction to induce cell apoptosis.

In summary, the research findings suggest that polysaccharides have the potential to induce cell apoptosis through the ROS/lysosome pathway, mitochondrial cascade reactions, and autophagy (Figure 2). However, further research is required to validate these findings and gain a better understanding of the underlying mechanisms involved in these processes. Additionally, while we have made progress in understanding how polysaccharides like *Hericium erinaceus* polysaccharides and fucoidan can affect cancer cell behavior, the challenge lies in maximizing the utilization of these properties to design effective anticancer treatment strategies. This remains an unresolved issue that requires further exploration.

### 4.3 Other pathways induced by polysaccharides

Polysaccharides have garnered significant attention for their unique anti-inflammatory, antioxidant, and antibacterial properties, especially in the realm of cancer therapy. They play a substantial role in modulating immune responses, protecting cells from oxidative stress damage, and inhibiting microbe-related pathological states (18). One key feature of polysaccharides is their anti-inflammatory effect. Studies have shown that polysaccharides derived from *Hovenia dulcis* fruits effectively influence the production and release of inflammation-related cytokines (TNF-α, IL-1, and IL-6), inhibit iNOS, and regulate oxidative stress, thereby suppressing inflammation associated with cancer progression (129, 130). This anti-inflammatory effect is crucial for preventing chronic inflammation related to cancer and deterioration of the cancer microenvironment.

Furthermore, oxidative stress is recognized as a significant contributing factor to various chronic diseases, including cancer. Polysaccharides from *Ginkgo biloba* leaves (GBPS) exhibit remarkable activities in scavenging hydroxyl and ABTS free radicals, enhancing antioxidant enzyme activities, and preventing oxidative damage to cellular DNA and other molecules, thus providing an effective defense mechanism (131, 132). Additionally, the antibacterial properties of polysaccharides also show potential in cancer treatment. They help inhibit the development and progression of cancer by affecting the microbial environment and interactions between microbes and host cells. Furthermore, crude polysaccharides from Chinese yam (57), *Angelica sinensis* polysaccharide (ASP) (10), ginger polysaccharides (133), and *Atractylodes macrocephala* polysaccharides (134) exhibit noteworthy anti-inflammatory, antioxidant, and antibacterial activities.

The adhesion, invasion, and metastasis of cancer cells are key factors in tumor aggravation and poor cancer prognosis. Some significant clinical studies have revealed that polysaccharides inhibit these processes by intervening in the interactions between cancer cells and their surrounding microenvironment (135). *Ganoderma lucidum* polysaccharides, by reducing the expression of β1 integrin on the surface of cancer cells, have demonstrated the ability to inhibit the adhesion of MT-1 cancer cells (136). Additionally, these polysaccharides can downregulate matrix metalloproteinase-9 (MMP-9) (18), hindering their capacity to invade adjacent tissues and migrate to distant organs. This intervention is critical for preventing the spread and metastasis of cancer. Furthermore, polysaccharides can regulate signaling pathways associated with cancer cell migration. In a colitis mouse model, modified Fuji apple polysaccharide (MAP) alters the LPS/TLR4/NF-κB pathway, significantly reducing the expression of LPS-induced TLR4, cyclooxygenase-2, matrix metallopeptidase 9 (MMP9), matrix metallopeptidase 2, inducible nitric oxide synthase, and prostaglandin E2 (137). This regulatory effect is not limited to direct action on cancer cells but can also impact the tumor microenvironment. *In vivo* studies have shown that polysaccharides EPS-1, extracted from the fermented Zygomycete filamentous fungus, possess the capability to inhibit tumor angiogenesis and metastasis in CT26 cells and lung tissue. This effect is achieved by suppressing the expression of vascular endothelial growth factor (VEGF), microvessel density (MVD), and platelet-derived growth factor (19), further limiting the nutritional supply and dissemination capacity of cancer cells (Table 1).

**Table 1:** The anti-colon cancer effects of polysaccharides.

Source	Polysaccharide	Monosaccharide composition	Mw (Da)	Glycosidic bond	Involved mechanism	Model	Reference
Brown algae (*Sargassum horneri*)	*Sargassum horneri* polysaccharides (SHP30)	Rhamnose	1.58 × 10^3^ kDa	/	Anti-tumor, inhibit cell cycle arrest and antioxidative	DLD cell	(32)
Longan (*Dimocarpus longan* L.)	Longan polysaccharides (LP)	Arabinose mannose, glucose and galactose	/	(1, 4)-β-glucose (1, 6)-β-mannose	Induce apoptosis by up-regulation of ROS	HT-29 cells	(33)
Yam (*Dioscorea opposita* Thunb.)	Chinese yam polysaccharides (CYP)	β-1,3-glucose, α-1-galactose, and α-1,6-galactose	16,619 Da	α-D-(1,4)-glucans	Stimulate immunity	Female ICR mice	(36)
*Atractylodes macrocephala* Koidz	Selenylated *Atractylodes macrocephala* polysaccharides (sAMPs)	The selenium content of sAMP4 was 12.23 mg g^−1^	/	α-(1, 4)-glucans β-(1, 4)-glucans	Enhance immunomodulatory activity	/	(35)
	RAMPtp	Mannose, glucose, rhamnose, galactose, and arabinose	/	β(1, 3) galactosidic linkage β(1, 6) galactosidic linkage	A novel lncRNA ITSN1OT1 was induced to maintain intestinal barrier function	IPEC-J2	(47)
	*Atractylodes macrocephala* polysaccharide (AMP)	Mannose: glucuronic acid: glucose: arabinose = 12.05: 6.02: 72.29: 9.64	2.391 × 10^4^ Da	α-(1, 4)-glucans β-(1, 4)-glucans	Regulates the metabolism of intestinal microbes to produce SCFAs	DSS treated mice	(89)
	*Atractylodes macrocephala* polysaccharide (AMP)	/	/	/	Anti-inflammation and antioxidant	RAW 264.7 murine macrophage cells	(134)
*Morus alba*	Mulberry leaf polysaccharides (MLP-2)	Mannose: rhamnose: glucose: galactose: arabinose = 1.31: 8.45: 6.94: 1.00: 11.96	2.22 × 10^6^ Da	β-D-pyranosidic linkage	Regulate immune function by improving the ND serum antibody titer and IL-2. Protect intestinal mucosa and promote slgA secretion	ICR mice	(56)
*Morchella esculenta*	*Morchella conica* polysaccharides (MCP)	D-mannose: D-glucose: D-galactose: L-rhamnose = 43.15: 19.56: 20.25: 1	3.974 × 10^3^ kDa	β-pyranosidic linkage	Regulate the immune function via NF-kB pathway. Reduced immune-suppression caused by cyclophosphamide. Changes the composition of intestinal flora	Cy-treated mice	(70)
*Rhizopus nigricans*	*Rhizopus nigricans* polysaccharides (RPS-1)	Glucose	1.617 × 10^7^ Da	α-(1, 4)-glucans	Stimulate immunity	CT26 tumor-bearing mice	(69)
	Exopolysaccharides1-1 (EPS1-1)	Glucose: galactose: mannose: fructose = 5.89: 3.64: 3.2: 1	9,682 Da	β-(1,6)-D-glucan	Suppressed the migration, invasion and adhesion abilities of cancer cells	CT26 tumor-bearing mice	(19)
*Hericium erinaceus*	*Hericium erinaceus* polysaccharides (HEPs)	Total carbohydrate 53.36% uronic acid 32.56%.	/	β-(1,3)-D-glucan β-(1,6)-D-glucan	Stimulate immunity via NF-кB, MAPK, PI3K/Akt pathway	Caco-2 cells	(65)
	HEFP-2b	Fucose: galactose: glucose: mannose = 11.81: 22.82: 44.28: 21.09	3.252 × 10^4^ Da	α-(1,6)-D-Glcp β-(1,4)-D-Galp α-(3,6)-D-Manp (1,6)-β-D-Galp (1,4)-α-D-Manp	Induce cell cycle arrest in S phase	HCT-116 cells	(105)
	Fruiting body polysaccharides of *Hericium erinaceus* (HEFPs)	Arabinose: galactose: glucose: mannose = 8.99: 11.15: 1.2: 1.97	/	α-(1,6)-Galp	Induce apoptosis via ROS	HCT-116 and DLD1 cell	(111)
*Ganoderma lucidum*	*Ganoderma lucidum* polysaccharides (GLP)	Arabinose: mannose: glucose: galactose = 4.19: 15.69: 78.15: 1.97	25.0 kDa	β-(1,3)-D-glucan β-(1,4)-D-glucan β-(1,6)-D-glucan	Reshaping microbial diversity and composition	Macrophage RAW264.7, intestinal HT-29, NCM460 cells	(82)
	GLP	Arabinose: galactose: glucose: cellulose = 11: 3: 3: 1	10–30 kDa, 32.1% 30–50 kDa, 21.8% >50 kDa, 46.1%	β-(1,3)-D-glucan β-(1,4)-D-glucan β-(1,6)-D-glucan	Induce apoptosis by mitochondrial pathway	LoVo cells	(110)
Brown algae (fucoidan)	Fucoidan	/	/	β-(1,4)-glucan	G1 phase cell cycle arrest	HT-29 cells	(106)
	Fucoidan	Galactose, xylose and mannose	/	β-(1,4)-glucan	Apoptosis is induced by ROS	Caco-2	(117)
	Fucoidan	Glucose: xylose: mannose: arabinose: galactose: fucose = 5.45 ± 2.97: 2.62 ± 2.12: 3.58 ± 1.34: 3.4 ± 1.39: 5.94 ± 3.88: 67.4 ± 12.1	1,200 Kda	β-(1,4)-glucan	Induce apoptosis binding by VC triggers	HCT-116	(128)
	FPS1M	Fucose: sulfate content =29.26: 6.41	130 kDa	(1,3)-α-L-fucopyranose (1,4)-α-L-fucopyranose	Enhance the sensitivity of chemotherapy	HCT116 cells and RKO cells	(21)
Red alga (*Porphyra haitanensis*)	*Porphyra haitanensis* polysaccharides (PHPs)	Total sugar: sulfate: uronic acid = 60.82 ± 1.56%: 9.02 ± 0.57%: 7.66 ± 1.54%	3.5 kDa	β-(1,3)-glucan	Induce apoptosis by increase the level of ROS	HT-29 cells LoVo cells SW-480 cells	(116)
Green tea	Tea polysaccharides (TPs)	Rhamnose: ribose: arabinose: mannose: glucose: galactose = 1.26: 3.18: 4.08: 1.00: 1.52: 3.92	28 kDa	α-(1,4)-glucan	Induce apoptosis via lysosomes—mitochondria pathway	CT26 cells	(120)
*Lentinus edodes*	Lentinan (SLNT)	Entirely of D-glucose	507.2 kDa	β-(1,3)-glucan	Induce autophagy death and apoptosis by endoplasmic reticulum stress pathway	HT-29 cells	(124)
*Laminaria digitata*	Laminarin	/	/	β-(1,3)-glucan β-(1,6)-glucan	Inhibits cancer cell growth by ErbB signaling pathway	HT-29 colon cancer cells	(127)
*Ginkgo biloba* L.	*Ginkgo biloba* leaves polysaccharides-2 (GBPS-2)	Mannose: rhamnose: glucuronic acid: galacturonic acid: glucose: galactose: arabinose: 0.08: 0.12: 0.16: 0.06: 0.11: 1.00: 0.32	672 kDa	α-D-glucan β-D-glucan	Stimulate immunity and antioxidant activity	RAW264.7 cell	(132)
*Angelica sinensis* (Oliv.) Diels	*Angelica sinensis* polysaccharide (ASP)	Fucose: galactose: glucose: arabinose: rhamnose: xylose 1.0: 13.6: 15.0: 8.7: 21.3: 3.7	Between 3.2 and 2,252 kDa	(1,4)-α-D-glucan (1,6)-α-D-glucan	Regulate immune function by activate macrophages to produce cytokines that induce the Th1/Th2 immune response and antioxidant, anti-inflammation activity	ICR mice	(10)
Ginseng (*Panax ginseng* Meyer)	Ginseng berry polysaccharide portion (GBPP)	/	76 kDa		Regulate immunity and reduce dose-related toxicity of 5-FU	HCT-116 and HT-29 CRC cell	(20)

## 5 Clinical strategies for the combination of polysaccharides and chemotherapy

In terms of *in vivo* and *in vitro* experimental data, natural polysaccharides hold promise as a potential viable therapy for colorectal cancer. When combined with chemotherapy, radiotherapy, or surgery, these polysaccharides have demonstrated improved therapeutic efficacy. For example, when used in conjunction with chemotherapy, polysaccharides not only exhibit synergistic anti-tumor effects but also reduce the occurrence of adverse reactions when used alone (138, 139). This opens up new perspectives and possibilities for the comprehensive treatment of colorectal cancer.

*In vivo*, studies have provided evidence that specific polysaccharides, such as ginseng berry polysaccharides (GBPP) can enhance the anti-cancer activity of 5-fluorouracil (5-FU) to varying degrees. This may be related to the downregulation of Th1 and Treg differentiation, and the regulation of immune tolerance (20). Additionally, chemotherapy drugs can damage the host’s immune response, polysaccharides extracted from wild morels (MCP) (70) and *Angelica sinensis polysaccharide* (ASP) (10) can increase the counts of leukocytes and lymphocytes in peripheral blood and spleen, and restore spleen weight in mice treated with chemotherapy, highlighting their potential as immunomodulators in chemotherapy. Some *in vitro* studies suggest that FPS1M (a laminarin-type polysaccharide extracted from brown seaweed) can enhance macrophage glycolysis and promote their polarization towards the M1 phenotype by activating the TLR4-mediated PI3K/AKT/mTOR signaling axis (21). This improvement in the tumor immune microenvironment enhances the sensitivity of colon cancer to capecitabine, thereby enhancing chemosensitivity. These *in vitro* research findings further confirm the significance of polysaccharides in their anticancer and immune-enhancing effects and demonstrate their crucial role in improving the tumor immune microenvironment.

These results are of utmost importance in understanding the mechanisms by which polysaccharides improve the tumor microenvironment and enhance chemosensitivity. However, the specific mechanisms of interaction between polysaccharides and chemotherapy drugs still remain elusive. Furthermore, it is crucial to emphasize the importance of translating these laboratory research findings into clinical applications. This will necessitate further clinical trials and studies to guarantee the safety and efficacy of these emerging treatment approaches.

## 6 Conclusion

The high incidence and poor prognosis of colorectal cancer present a significant challenge to global health. Traditional surgical and chemotherapy treatments have not yielded satisfactory results in combating this disease. In this study, we highlight the tremendous potential of natural polysaccharide extracts in the treatment of colorectal cancer. Derived from natural sources, these polysaccharides exhibit a wealth of pharmacological activities, low toxicity, and high biocompatibility, making them a valuable addition to colorectal cancer research. Preclinical trials have demonstrated that these polysaccharides may treat colorectal cancer by protecting the intestinal epithelial barrier, enhancing the immune system, modulating the gut microbiota, and exerting anti-tumor, antioxidant, and anti-inflammatory effects. When used in conjunction with chemotherapy drugs, these polysaccharides can also reduce the side effects of chemotherapy, offering greater benefits to patients. This study elucidates the specific mechanisms of polysaccharide treatment in colorectal cancer and provides a comprehensive review of recent research advancements.

While current research has yielded encouraging insights into the use of polysaccharides for cancer treatment, several critical issues remain to be addressed. Firstly, the efficacy of polysaccharides as oral drugs must be assured. The diverse sources of these polysaccharides introduce complexity in quality and consistency control, which can be mitigated through enhanced extraction techniques to achieve standardization. However, the challenge of targeting specific disease sites with polysaccharides may reduce therapeutic effectiveness or increase the risk of side effects. To counter this, the integration of advanced delivery systems such as nanotechnology or chitosan is proposed to enhance the targeting and efficacy of the drugs. Secondly, existing experimental results are primarily confined to *in vitro* studies or mouse models. Considering the complexity of the human body and tumor microenvironments, further clinical trials are imperative to validate the real-world efficacy of polysaccharides. These trials should explore unknown anti-cancer effects, potential side effects, and resistance issues. Additionally, variability in patient responses may lead to inconsistent treatment outcomes. Future research strategies should focus on improving drug delivery systems to enhance the bioavailability of polysaccharides, combining various treatment modalities to increase effectiveness, implementing personalized medical approaches, and conducting extensive research and clinical trials for a deeper understanding of the role of polysaccharides in cancer therapy. Comprehensive safety assessments of polysaccharides and close monitoring of patients during treatment are crucial to ensure safe and effective use.

Thirdly, interactions between polysaccharides and other dietary components or drugs in the gastrointestinal tract could affect their release and absorption. Therefore, the palatability and patient acceptability of polysaccharides as oral drugs are key considerations. Lastly, investigating the molecular mechanisms of polysaccharide anti-cancer activity is vital. Although current studies have identified some interactive signaling pathways and potential active components of polysaccharides, the specific targets of their interaction with host and tumor cells require further clarification. Research should also focus on the specific pathways of action and the synergistic effects with other treatment methods. By employing these comprehensive approaches, we can overcome the limitations of polysaccharides in cancer treatment and enhance their clinical application value.

## Author contributions

JF: Conceptualization, Data curation, Writing – original draft. JZ: Supervision, Validation, Visualization, Writing – review & editing. HZ: Supervision, Validation, Visualization, Writing – review & editing. YZ: Supervision, Validation, Visualization, Writing – review & editing. HX: Supervision, Validation, Visualization, Writing – review & editing.
